# Comparative Analysis of the Spectral Response to Soil Salinity of Saline-Sodic Soils under Different Surface Conditions

**DOI:** 10.3390/ijerph15122721

**Published:** 2018-12-03

**Authors:** Jianhua Ren, Kai Zhao, Xiangwen Wu, Xingming Zheng, Xiaojie Li

**Affiliations:** 1 Heilongjiang Province Key Laboratory of Geographical Environment Monitoring and Spatial Information Service in Cold Regions, Harbin Normal University, Harbin 150025, China; renjianhua_hrb@163.com (J.R.); wuxiangwen_hrb@163.com (X.W.); 2 Northeast Institute of Geography and Agroecology, Chinese Academy of Sciences, Changchun 130102, China; zhaokai@neigae.ac.cn (K.Z.); zhengxingming@neigae.ac.cn (X.Z.)

**Keywords:** desiccation cracking, saline-sodic soils, soil salinization, spectral response

## Abstract

Desiccation cracking is a very common surface soil phenomenon of saline-sodic land. The objective of this study was to investigate the effects of salt content on the spectral reflectance of soil with and without desiccation cracks. To achieve our objective, a cracking test was performed using 17 soil samples. Following the tests, crack parameters were extracted, and correlation analysis was then performed between crack parameters and four soil properties: Na^+^, salinity (total concentration of ions), pH, and electric conductivity (EC). In order to select the optimum spectral measurement method and develop prediction models, spectral response to different soil properties were compared between the cracked soil samples and the comparative soil samples composed of the 2 mm particle size fraction processed by traditional methods. The results indicate that soil salinity dominated cracking propagation with a positive correlation. Since area and volume scattering are closer to what occurs in the field, a greater spectral response to soil properties was found for cracked soil samples relative to the comparative soil samples in the near-infrared and shortwave-infrared regions. The *R*^2^ of optimal linear prediction models based on the cracked soil samples were 0.74, 0.67, 0.58, and 0.67 for Na^+^, salinity, pH, and EC, respectively.

## 1. Introduction

Soil salinization is a severe land degradation process which results in severe impairment to plant/crop growth and development, reducing agricultural productivity. The condition is usually found in arid, semi-arid regions, and along coastal soils, presenting serious risk to regional economies and the environment. Nearly 9.5 × 10^7^ hectares of area worldwide are estimated to be salt-affected, which is more than 7% of the Earth’s continental surface [1]. In addition, soil salinization is expected to increase as the human population increases and the industry develops in the future [2]. Thus, it is very important to identify salt-affected areas and determine their composition, to enable remediation of saline and saline-sodic soils.

Due to the ability of remote sensing techniques, remote sensing has been widely used to identify salt-affected soil, map saline areas, and estimate salinity levels [3]. Several studies have indicated that saline soils have distinctive spectral features in the near-infrared and shortwave-infrared regions. Taylor et al. [4] studied spectral features of saline soils and found strong reflection peaks at 800 nm and absorption valleys at 980, 1170, 1450, and 1900 nm. Farifteh et al. [5] recorded similar reflectance measurements for salt-affected soils. Their results showed significant correlations between soil EC and spectral measurements, indicating that similarity between sample spectra decreased as the salt concentration increased. Weng et al. [6] quantitatively analyzed the relationship between spectral characteristics and salinity of saline soils, and found that for soil with high NaCl and MgCl_2_ content, there was a negative correlation between salinity and spectral reflectance, from 1931 nm to 2123 nm, and a positive relationship from 2153 nm to 2254 nm. Nawar et al. [7] took the differentiated and declustered spectral reflectance of saline soils in the El-Tina Plain, Egypt, as spectral parameters, and calculated the correlation coefficient between the difference index (DI), the normalized difference index (NDI), and the ratio index (RI) of the DI and NDI parameters and EC. The results showed that the first-order differential form of reflectance had the greatest correlation with EC at 1843 nm and 1918 nm, with an *R*^2^ of 0.65. Moreira et al. [8] amended soil to provide samples of a range of salinity using NaCl, MgCl_2_, and CaCl_2_. They measured the spectral features of the amended soil and found absorption bands of 1470, 1750, and 2200 nm, characteristic of salinity. Their study also showed the spectral reflectance decreased with the CaCl_2_ and MgCl_2_, while NaCl-amended soil showed a reverse trend.

Srivastava et al. [9] developed robust reflectance spectral models for rapid assessment of soil salinity in the salt-affected areas of Indo-Gangetic plains of Haryana using VNIR (visible-near infrared) reflectance spectroscopy. The spectral models explained more than 80% of EC variability in EC, and additional salinity-related properties. Zhang et al. [10] developed a soil salt content model using the fractional-order derivatives of field-measured spectral data paired with ground measurements. They found that there was a significant correlation between single band reflectance spectra and soil salt content. As the derivative order increased, reflectance values first increased, and then decreased, with a peak *R*-value of 0.525 in the 1.2-order derivative.

Dehaan and Taylor [11] used reflectance spectroscopy for recognition and classification of saline soil. The study related vegetation with irrigation-induced salinization, for characterizing and mapping the spatial distribution of salinization. Zhang et al. [12] combined spectral technology with multivariant statistical analysis to determine the reflectance spectral features of saline soil. They constructed quantitative regression models that considered the eliminated influence from instrument errors and other external background factors. Yang et al. [13] presented a similarity-based prediction approach to map soil salinity, and developed powerful environmental predictors for the Huanghe River Delta region in China. Their results showed that a similarity-based prediction approach was a viable alternative to other methods for mapping soil salinity, especially for mapping areas with limited observation data. Wang and Jia [14] integrated multisensor remote sensing data with a field survey to analyze processes of soil salinization in a semi-arid area in China from 1979 to 2009. Their results showed that the area of salt-affected soils increased by 0.28% per year with remarkable acceleration increase. Wang et al. [15] combined the arid fraction integrated index (AFII) and salinity index (SI), and developed an operational model to estimate the surface soil salt content. Their results indicate that the model can provide information on soil salinity with exponential relationships between soil salinity and the model with *R*^2^ > 0.86 and RMSE < 6.86. Additional spectral analysis techniques, such as spectral mixture analysis, were also used to predict soil salinity [16,17,18]. The statistical techniques of single variable linear regression, polynomial regression, principle component regression, partial least square regression (PLSR), artificial neural networks (ANN), and multiple adaptive regression splines (MARS), have also been used to recognize soils with different salinity levels, and illustrate the relationships between soil spectral reflectance and soil properties [19,20,21,22,23,24,25].

Desiccation cracking of saline-sodic soils is a very common surface soil phenomenon after precipitation. Cracking during soil drying is controlled by physical properties, including soil suction, tensile stress, shear strength, tensile strength, and specific surface energy [26,27,28,29,30,31]. In most cases, these physical properties are dependent on clay content and clay species. Soil salinity also alters soil cracking properties to a great extent. After a series of studies on 32 saline-alkali soils, Smith et al. [32] determined the relationship between the cation exchange capacity of the soils and their linear expansion coefficient (COLE), with a correlation coefficient up to 0.81. Ren et al. [33,34] extracted the textural features of crack patterns, and quantitatively analyzed the relationship between texture feature and salinity of saline-sodic soils. Zhang et al. [35] conducted desiccation tests of saline-sodic soils in the laboratory to analyze the effects of NaCl content on their cracking behavior during water evaporation. They showed that as the NaCl content increased, the crack intensity factor value and the crack length decreased. Shokri et al. [36] and Zhang et al. [37] studied the effects of salt content on the diffuse double layer (DDL) of saline soils. They found that DDL plays a very important role in controlling the shrinking and cracking process of saline soils during drying. Since desiccation crack changes the surface conditions of saline-sodic soils in Songnen Plain, and further influences the radiative transfer process of spectral reflectance and the area scattering within the cracks, various crack conditions of soils with different salinity levels may, therefore, affect the spectral response of soils to a certain extent. In most laboratory studies, traditional spectral measurements are used to examine soil samples after grinding over 2 mm sieve, but rarely take into account the natural conditions of soil surface of saline-sodic soils which develop cracks during drying. In this study, laboratory cracking experiments were carried out in order to examine, characterize, and compare the spectral response of saline-sodic soils, with and without desiccation cracks, and then regression models were developed for prediction of their saline-sodic properties.

## 2. Materials and Methods

### 2.1. Study Sites

The Songnen Plain contains one of the three areas in the world with the largest area of saline-sodic soils. The main salt components of NaHCO_3_ and Na_2_CO_3_ make the salt-affected soils in Songnen Plain saline-sodic in nature [38]. The saline-sodic soils generally have a similar clay-loam texture, with the original mineral of quartz and feldspar, and secondary mineral of illite/smectite formation, which have the characteristic of forming cracks when drying [39]. However, the cracking process usually occurred on the soil surface, since the saline soil layer at the depth of 20–80 cm is a typical columnar deposition, which has poor water infiltration properties and prevents downward movement of salt [40,41,42,43]. In this study, 17 soil samples were therefore obtained, from 0 to 20 cm. All the soil samples were collected using a spade within a very narrow area of Da’an City, Songnen Plain, west of Jilin Province, China (123°47’35’’ E to 123°59’55’’ E, 45°29’30’’ N to 45°36’26’’ N). The soil samples were air-dried (temperature of 25 °C, humidity of 35%), ground, passed through a 2 mm sieve, and then divided into three subsamples for independent analysis. The first subsamples were prepared for measurement of soil properties in the laboratory; the second subsamples were prepared as the cracked soil samples undergoing the desiccation cracking test for spectral collection; the third subsamples were completely dried in an oven, and prepared as the comparative soil samples (soil particle size of 2 mm) for spectral collection. 

### 2.2. Soil Property Measurement

All chemical properties of the soil samples were analyzed in a laboratory. The main ions of interest, Na^+^, K^+^, Ca^2+^, Mg^2+^, Cl^−^, HCO_3_^−^, and CO_3_^2−^ were extracted using a water extract with a soil–water ratio of 1:5. The Na^+^ and K^+^ were measured using a flame photometer; Ca^2+^ and Mg^2+^ were analyzed using the EDTA complexometric titration method; Cl^−^ was measured by the AgNO_3_ titration method; CO_3_^2−^ and HCO_3_^−^ were measured using the double indicator neutralization method. The soil pH and EC were analyzed with the potentiostatic method and conductometry respectively, using soil suspensions with a soil–water ratio of 1:5. The particle size distributions of the soil samples were also determined using laser diffraction analysis method. 

### 2.3. Desiccation Cracking Test

**Figure 1:**
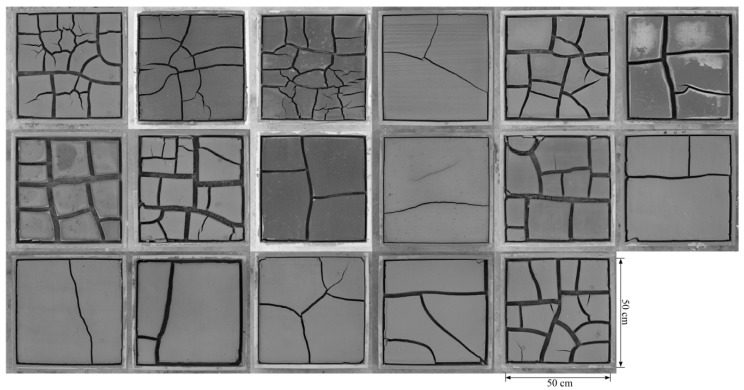
The cracked soil samples after the drying process.

In order to produce crack patterns in the laboratory, saturated slurries were prepared with 80% gravimetric water content. The soils were slurried by hand-mixing, and were poured individually into a box with a size of 50 cm × 50 cm × 3 cm, and the surface was smoothed with a spatula. Afterwards, the soil samples were subjected to controlled laboratory conditions (temperature of 25 °C, humidity of 35% and pressure of 101 kPa) from 11 September 2014 to 3 October 2014, until the desiccation cracking process was completed (all weights of the samples stabilized, Figure 1), and the final water content of all the cracked soil samples were then measured.

**Figure 2:**
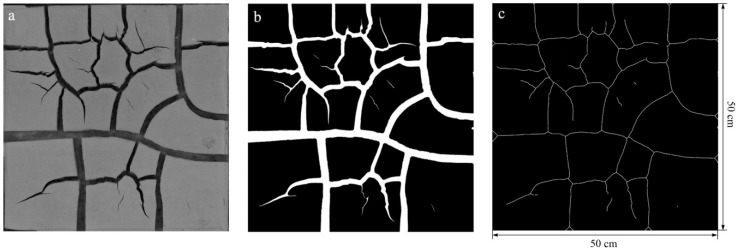
The digital image processing of crack patterns.

A digital camera was installed on a fixed experimental platform to determine the morphologies of the cracked soil samples, and a standard colorimetric plate was used to calibrate the white balance of the camera to reduce the effect of changes in the photographic environment. Digital image processing was then used to extract the characteristics of crack patterns, and the procedure is shown in Figure 2. First, the color photo was cropped and converted to a grayscale image (Figure 2a). Second, the grayscale image was segmented into cracks (white areas) and aggregates (black areas) using a gray threshold after binarization (Figure 2b). Third, a skeletonization algorithm was utilized to repeatedly remove pixels from the boundaries of the cracks, until one-pixel-wide skeletons remained (Figure 2c). After that, the crack length (CL) was determined by counting pixels based on the binary skeletons of the crack patterns, the crack area (CA) was extracted by computing the number of white pixels representing them, and the crack ratio (CR) was also calculated to quantify the cracking extent.

### 2.4. Spectral Measurements and Comparative Analysis

In order to obtain the spectral characteristics, an SVC HR-1024 hand-held spectrometer was used in this experiment. This spectrometer is sensitive to wavelengths between 350 and 2500 nm, and includes three separate detectors; the VNIR (350–1000 nm); the SWIR1 (short-wave infrared 1, 1000–1850 nm); and the SWIR2 (short-wave infrared 2, 1850–2500 nm). The sampling intervals were 1.5 nm with a spectral resolution of 3.5 nm, 3.6 nm with a spectral resolution of 6.5 nm, and 2.5 nm with a spectral resolution of 6.5 nm, for wavelengths 350–1000 nm, 1000–1850 nm, and 1850–2500 nm, respectively. 

In this investigation, specific spectral measurements were based on both the cracked soil samples and the comparative soil samples. For the cracked soil samples, a fiber optic with a 25° field of view was held on a fixed platform 1 m above the ground (corresponding to a circle field with a diameter of 45 cm), a 50 cm × 50 cm rectangle region was then determined on the ground to make the center of this region coincide with the lens centerline projection of the fiber optic to ensure that both the zenith angle and the azimuth angle were same for all the cracked soil samples. After that, each cracked soil sample was placed in the rectangle region for spectral measurement. For the 2 mm comparative soil samples, a 4° field of view was chosen 20 cm vertically above the samples. Note that the comparative samples were put separately into the same aluminum boxes with an inner diameter of 10 cm and a depth of 2 cm. For each spectral measurement, 10 readings of spectral reflectance were taken, and the mean of all spectral reflectance was calculated as the actual reflectance of the sample, in order to average out the difference caused by instrument noise. A standard white panel was also measured before each measurement as calibration. In order to diminish the noise of the equipment and reduce the data dimensions, all the spectral reflectance of soil samples was resampled at 10 nm. The reflectance ranges of 1350–1420 nm, 1800–1920 nm, and 2360–2500 nm were cut off for all the spectral measurements, due to the effects of water absorption bands. Considering the sensitivity to the spectral variations and the background noise of reflectance, typical mathematical transformations of reflectance, including the square root (*R*^1/2^) and the logarithm (Lg*R*), were computed in this study, and they were then treated together with reflectance as spectral parameters in this study. The spectral reflectance characteristics of soil samples with different crack extents were then analyzed and compared with that of the 2 mm comparative soil samples. Afterwards, the correlation coefficients between the Na^+^, salinity, pH, EC, and spectral parameters were computed through the whole bandwidth for all the cracked soil samples and the comparative soil samples.

## 3. Results

### 3.1. Soil Properties

Table 1 presents soil properties for the 17 soil samples. All soils were alkaline, with pH from 8.76 to 10.60, the EC ranges of the soil samples are quite various from 0.09 ds/m to 3.00 ds/m. Na^+^ is the dominant cation in the soil samples, with a pH >9.5, and Ca^2+^ and Mg^2+^ are the main cations with a pH <9.5, while HCO_3_^−^ is the main anion.

Table 2 shows the soil particle distributions of the soil samples. It can be seen that the range of clay is very small (from 26.28% to 29.37%), that the standard deviation (SD) and the coefficient of variation (CV) are only 1.42 mg/g and 4.36%, indicating that no significant differences were observed in the clay content for the soil samples.

### 3.2. Crack Parameters

Table 3 provides the results for crack parameters of the cracked soil samples. Crack parameters between soils were quite different, with CL ranges from 61.50 to 397.92 cm, CA ranges from 23.94 to 478.21 cm^2^, and CR from 1.01% to 21.96% respectively, and the trends of all the crack parameters were quite similar. Note that all crack parameters were calculated within the central circle region with a diameter of 45 cm by setting null values to the other regions of the image of each cracked soil sample in order to match spectral measurements. 

### 3.3. Spectral Characteristics

**Figure 3:**
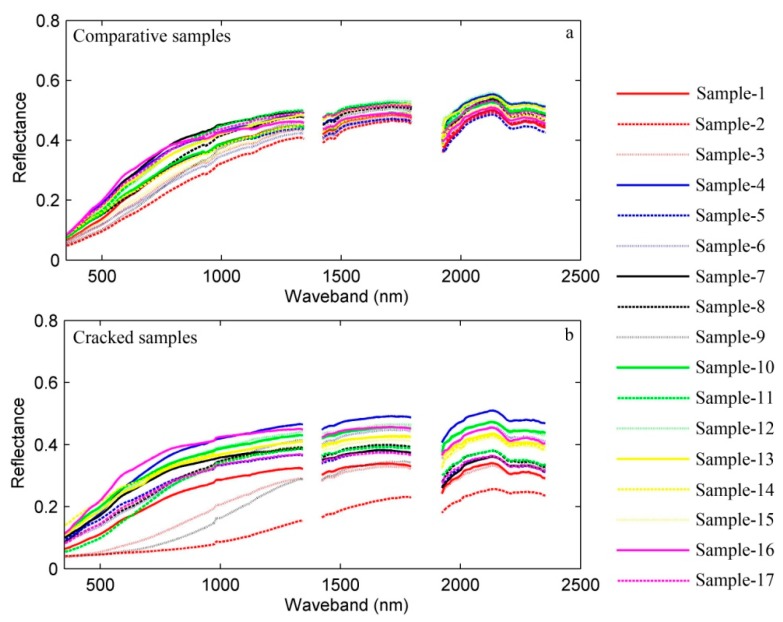
Spectral reflectance curves of soil samples with different salinity levels.

The spectral reflectance curves of all the cracked soil samples and the comparative soil samples with variable salinity levels are shown in Figure 3. All comparative soil samples shared similar curvature in Figure 3a, yet differences between samples can be identified at wavebands from 600 to 1000 nm. In this region, some soils had slight absorption features, while slight reflection peaks were presented in other curves. However, Figure 3b shows quite different curve shapes for the cracked soil samples in the visible and near-infrared region below 1350 nm. In this region, some cracked soil samples had significant absorption features, while others have reflection peaks. Figure 3b also shows that the overall reflectance curves of all the cracked soil samples were lower than the reflectance curves of the comparative soil samples in Figure 3a, and the differences among reflectance curves of all the cracked soil samples were more apparent compared with those of the comparative soil samples.

### 3.4. Correlation Analysis

**Figure 4:**
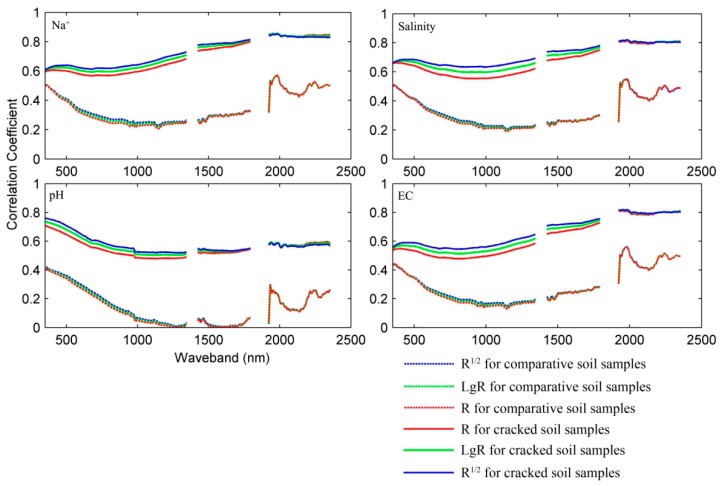
Correlograms between original spectral parameters and soil properties.

In order to illustrate the relationships between spectral parameters and soil properties of all soil samples for extracting the optimum spectral parameter and the corresponding diagnostic wavelength, correlograms were calculated across four soil properties of particular interest in this study (Na^+^, salinity, pH, and EC) for spectral parameters of all the cracked soil samples and the comparative soil samples. They display absolute value curves of correlation coefficient between spectral parameters, and a given soil property as a function of wavelength (Figure 4). The curves of spectral parameters shared nearly the same shapes for all cracked soil samples, and the correlograms for the comparative soil samples also shared similar shapes. The *R*^1/2^ and Lg*R* transform curves were slightly higher than the *R* curve, especially for the cracked soil samples in the region under 1800 nm, due to their sensibility enhancement for reflectance. The curves of the cracked soil samples were much higher than the comparative soil samples. Similar tendencies of the correlogram curves for Na^+^, salinity, and EC are also evident. The pH of the soils shared a similar shape, but had a quite different slope and margin compared with the other properties. For the comparative soil samples, the curves for soil properties first dropped, and then rose to a maximum at about 1800 nm, with some minor fluctuations; for the cracked soil samples, correlation coefficient curves of Na^+^, salinity, and EC changed gradually, and reached stabilization at about 1430 nm. The correlation coefficient curves of pH decreased from approximately 350 to 1000 nm, and then remained stable at higher wavelengths.

## 4. Discussion

Table 4 shows the correlation coefficient matrix between the soil parameters of interest (Na^+^, salinity, pH, and EC) in this investigation, and indicates that the salinity and EC increase with Na^+^ concentration, since Na^+^ was the main cation in these soils.

Table 5 provides the correlation coefficients between the main soil properties and crack parameters. The results showed that the correlations were high between CL and saline properties. At correlation coefficients greater than 0.52, the correlations between CA, CR, and soil properties were also quite high.

Table 5 also indicates that the soil salinity dominated the extent of cracking propagation (CL, CA, and CR) for the soils. This is because all soil samples were saturated, with a water content of 80% at the onset of the study, combined water films were formed, generally, due to the interaction soil colloidal particles and exchangeable cations (especially Na^+^, with its large hydrolytic radius in this study) during the desiccation cracking process, and the thickness of the combined water films increase with soil salinity. The formed water films dispersed the cementation between the clays and increased the spacing of soil particles, resulting in reduced cohesive strength, which reflects the tensile strength between soil colloidal particles, and also reduced the internal friction angles between soil particles which reflect the soil shear strength [44]. On the other hand, the salt solution among soil particles can be regarded as a lubricant, and further decreases the internal friction angles between soil particles [45,46]. Table 6 shows the gravimetric water content of cracked soil samples after air-drying, where the mean value of final water content was 2.04 %. This indicates that the slight effects of water absorption caused the overall reflectance of cracked soil samples to be lower than those comparative soil samples completely dried in an oven. However, the small variance of the final water content indicates the decrease of reflectance by water absorption was nearly equal for all the cracked soil samples, which means that soil water content was not the factor that caused variation in spectral response in this study.

Figure 3 shows that the variations among reflectance curves of all the cracked soil samples were more apparent than those of the comparative soil samples. This is because the salt crystals present on cracked soil sample surfaces—after the drying process—varied, which means that the spectral response to these salt crystals were different among the cracked soil samples. Table 5 indicates that soil salinity leads to an increase of crack extent. The area scattering on soil surface and volume scattering within crack regions strengthened with the increasing relative surface roughness and the complexity of surface morphology determined by the crack extent, which reduced the energy reflected to the spectrometer, and thus enhanced the spectral differences between different cracked soil samples. In addition, the area and volume scattering from the cracked soil samples were also reasons for their lower overall reflectance than the comparative soil samples.

For the cracked soil samples, the correlation analysis results between spectral parameters and the main soil properties were better, and the spectral response were closer to the salt minerals compared with those of the comparative soil samples. This is because salt crystals formed at the surface and cracks of the cracked soil samples during desiccation, and that the salt crystal content increased with salinity. The spectral characteristics were, therefore, more apparent for the cracked soil samples than the comparative dried soil samples in near-infrared and shortwave-infrared regions above 1420 nm, which contain the most crucial information [47,48] for the salt minerals in this study. For pH, the relatively poor correlation analysis results are probably due to the incomplete hydrolysis of HCO_3_^−^ and CO_3_^2−^. Thus, the relationships between pH and spectral parameters were generally not significant above 1420 nm, but were more important in visible regions compared to Na^+^, salinity, and EC. 

**Figure 5:**
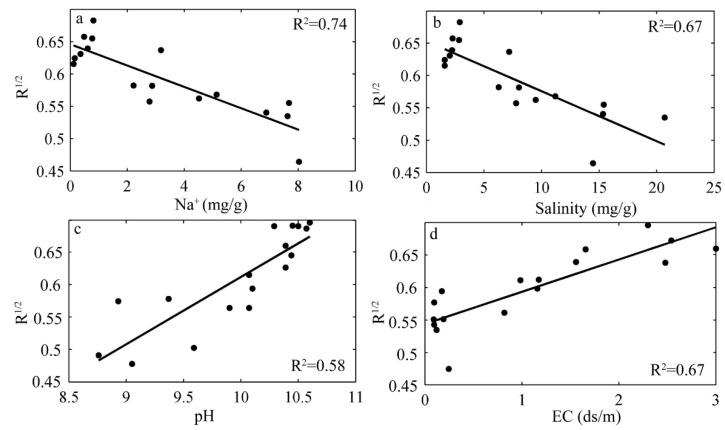
Regression models for soil properties of the cracked soil samples.

In order to generate improved prediction of Na^+^, salinity, pH, and EC, the optimum spectral parameter, the corresponding wavelength and the correlation coefficient (*R*-value) for each soil property was extracted from the correlograms for spectral parameters of the cracked soil samples and the comparative soil samples. Table 7 indicates the optimal *R*-value derived from cracked soil samples is obviously higher than that derived from the comparative soil samples. For Na^+^, salinity, and EC, the diagnostic wavelength was 1983 nm while, for pH, the diagnostic wavelength was 363 nm. The *R*^1/2^ was selected as the optimal spectral parameter, since it enhanced small variations in spectral reflectance, and was less sensitive to the spectral variations of sunlight and skylight. Also, *R*^1/2^ tended to eliminate background noise. The single-variable linear regression model for each soil property was, therefore, established and shown in Figure 5, indicating good prediction of the soil properties of interest in this study. 

## 5. Conclusions

This study performed a desiccation cracking experiment for 17 saline-sodic soil samples with different salinity levels from the Songnen Plain of China. It also compared the spectral response of cracked soil samples to that of 2 mm comparative soil samples for each soil property of interest. Linear regression models were developed for each main soil property through the optimum spectral parameter at the corresponding diagnostic waveband based on the cracked soil samples. The main conclusions in this investigation are as follows:

(1). Soil salinity dominates the cracking propagation extent with a quite positive correlation, provided that clay content and clay mineral composition between samples are similar.
(2). The cracked soil samples have higher correlation with soil properties compared with the comparative soil samples.
(3). The regression models show good predictive abilities for soil properties based on cracked soil samples for Na^+^, total salinity, pH, and EC.

However, there are still some limitations to be considered before the procedure is used generally to characterize saline-sodic soils. For instance, the effects of background (known as wooden) on spectra were ignored in this study, and the radiation transfer process of energy on the surface of the cracked soil and within the cracked regions was not very clear. Although the results of these experiments are encouraging, the predictive regression models require data verification.

## Figures and Tables

**Table 1:** Physicochemical properties of soil samples.

Sample	pH	EC (ds/m)	Na^+^ (mg/g)	K^+^ (mg/g)	Ca^2+^ + Mg^2+^ (mg/g)	HCO_3_^−^ (mg/g)	CO_3_^2−^ (mg/g)	Cl^−^ (mg/g)	Salinity (mg/g)
1	10.44	2.54	6.88	0.01	1.28	2.93	1.38	2.88	15.36
2	10.60	2.30	8.03	0.04	1.28	2.07	1.62	1.44	14.48
3	10.57	2.48	7.63	0.01	1.12	6.34	4.62	0.99	20.71
4	10.07	0.25	0.81	0.01	0.64	1.16	0.12	0.16	2.90
5	10.10	1.66	4.51	0.01	0.71	1.71	0.48	2.08	9.50
6	10.29	1.16	3.18	0.02	0.86	2.75	0.12	0.27	7.20
7	10.07	3.00	7.67	0.01	0.64	2.14	0.84	4.10	15.40
8	10.39	0.82	2.22	0.01	0.58	3.05	0.18	0.24	6.28
9	10.45	0.99	2.78	0.02	0.80	3.23	0.54	0.43	7.80
10	9.37	0.12	0.48	0.01	0.64	1.04	0	0.11	2.28
11	10.50	1.17	2.88	0.02	0.51	3.90	0.54	0.20	8.05
12	9.90	0.19	0.77	0.01	0.45	1.46	0	0.15	2.84
13	9.05	0.09	0.36	0.01	0.64	0.92	0	0.11	2.04
14	8.76	0.10	0.16	0.01	0.74	0.61	0	0.08	1.60
15	8.93	0.09	0.12	0.01	0.70	0.67	0	0.09	1.59
16	9.59	0.17	0.61	0.01	0.48	1.04	0	0.11	2.25
17	10.39	1.56	5.14	0.02	0.32	3.11	1.26	1.35	11.20

**Table 2:** Statistical description of the soil particle distributions of the soil samples.

Soil Texture	Min	Max	Mean	SD	CV (%)	Skewness	Kurtosis
Clay (%)	26.28	29.37	27.73	1.42	4.36	0.42	−0.25
Silt (%)	29.62	38.41	36.29	3.18	8.04	−0.13	−0.78
Sand (%)	28.26	40.13	37.84	3.62	9.82	−0.22	−0.81

SD standard deviation, CV coefficient of variation.

**Table 3:** Crack parameters of cracked soil samples.

Samples	CL (cm)	CA (cm^2^)	CR (%)
1	361.04	367.23	16.01
2	241.01	164.31	7.10
3	397.92	264.42	11.56
4	71.36	29.45	1.26
5	248.34	342.73	15.67
6	139.50	184.40	8.26
7	240.39	428.55	19.59
8	310.20	478.21	21.96
9	105.26	117.99	5.19
10	71.21	23.94	1.01
11	245.71	367.30	16.50
12	73.48	63.59	2.83
13	61.50	37.02	1.60
14	79.40	114.32	5.24
15	121.80	86.86	3.68
16	128.47	159.56	7.24
17	281.47	397.51	18.30

CL crack length, CA crack area, CR crack ratio.

**Table 4:** Correlation between the main soil parameters.

	Na^+^	Salinity	pH	EC
Na^+^	1			
Salinity	0.97	1		
pH	0.71	0.73	1	
EC	0.98	0.96	0.69	1

**Table 5:** Correlation coefficients between crack parameters and main soil properties.

Crack Parameters	Na^+^	Salinity	pH	EC
CL	0.80	0.86	0.68	0.81
CA	0.62	0.62	0.58	0.68
CR	0.60	0.60	0.57	0.66

**Table 6:** Statistical parameters for final gravimetric water contents of cracked soil samples.

Maximum	Minimum	Mean	Variance
2.32	1.83	2.04	0.03

**Table 7:** Optimal spectral parameters and corresponding wavelengths for different soil properties of soil samples.

Soil Property	Cracked Soil Samples			Comparative Soil Samples		
	Parameter	Wavelength (nm)	*R*-value	Parameter	Wavelength (nm)	*R*-value
Na^+^	*R* ^1/2^	1983	0.86	*R* ^1/2^	1983	0.57
Salinity	*R* ^1/2^	1983	0.82	*R* ^1/2^	1983	0.55
pH	*R* ^1/2^	363	0.76	*R* ^1/2^	363	0.41
EC	*R* ^1/2^	1983	0.82	*R* ^1/2^	1983	0.56

## References

[1] Ghassemi F., Jakeman A.J., Nix H.A. (1995). Salinization of Land and Water Resources: Human Causes, Extent, Management and Case Studies.

[2] Kicińska A., Kosa-Burda B., Kozub P. (2018). Utilization of a sewage sludge for rehabilitating the soils degraded by the metallurgical industry and a possible environmental risk involved. Hum. Ecol. Risk Assess.

[3] Mulder V.L., de Bruin S., Scheapman M.E., Mayr T.R. (2011). The use of remote sensing in soil and terrain mapping—A review. Geoderma.

[4] Taylor G.R., Hemphill P., Currie D., Broadfoot T., Dehaan R.L. (2001). Mapping dryland salinity with hyperspectral imagery. IEEE Int. Geosci. Remote Sens. Symp. Scanning Present Resolv. Future.

[5] Farifteh J., van der Meer F., Carranza E.J.M. (2007). Similarity measures for spectral discrimination of salt-affected soils. Int. J. Remote Sens..

[6] Weng Y., Gong P., Zhu Z. (2008). Reflectance spectroscopy for the assessment of soil salt content in soils of the Yellow River Delta of China. Int. J. Remote Sens..

[7] Nawar S., Buddenbaum H., Hill J. (2014). Estimation of soil salinity using three quantitative methods based on visible and near-infrared reflectance spectroscopy: A case study from Egypt. Arabian J. Geosci..

[8] Moreira L.C.J., Teixera A.S., Galvao L.S. (2014). Laboratory salinization of Brazilian alluvial soils and the spectral effects of gypsum. Remote Sens..

[9] Srivastava R., Sethi M., Yadav R.K., Bundela D.S., Singh M., Chattaraj S., Singh S.K., Nasre R.A., Bishnoi S.R., Dhale S. (2017). Visible-Near infrared reflectance spectroscopy for rapid characterization of salt-affected soil in the Indo-Gangetic Plains of Haryana, India. J. Indian Soc. Remote Sens..

[10] Zhang F., Wang X., Kung H.K., Johnson V.C. (2018). Estimating soil salt content using fractional derivatives and optional spectral indices in the Ebinur Lake Oasis, Northwestern China. Data-Enabled Discov. Appl..

[11] Dehaan R.L., Taylor G.R. (2002). Field-derived spectra of salinized soils and vegetation as indicators of irrigation-induced soil salinization. Remote Sens. Environ..

[12] Zhang F., Tiyip T., Ding J., Kung H., Johnson V.C., Sawut M., Tashpolat N., Gui D. (2013). Studies on the reflectance spectral features of saline soil along the middle reaches of Tarim River: A case study in Xinjiang Autonomous Region, China. Environ. Earth Sci..

[13] Yang L., Huang C., Liu G., Liu J., Zhu A.X. (2015). Mapping soil salinity using a similarity-based prediction approach: A case study in Huanghe River Delta, China. Chin. Geogr. Sci..

[14] Wang H., Jia G. (2012). Satellite-based monitoring of decadal soil salinization and climate effects in a semi-arid region of China. Adv. Atmos. Sci..

[15] Wang F., Luo G., Ding J., Chen X. (2013). Detecting soil salinity with arid fraction integrated index and salinity index in feature space using Landsat TM imagery. J. Arid Land.

[16] Masoud A.A. (2014). Predicting salt abundance in slightly saline soils from Landsat ETM+ imagery using Spectral Mixture Analysis and soil spectrometry. Geoderma.

[17] Fourati H.T., Bouaziz M., Benzina M., Bouaziz S. (2015). Modeling of soil salinity within a semi-arid region using spectral analysis. Arabian J. Geosci..

[18] Fourati H.T., Bouaziz M., Benzina M., Bouaziz S. (2017). Detection of terrain indices related to soil salinity and mapping salt-affected soils using remote sensing and geostatistical techniques. Environ. Monit. Assess.

[19] Volkan Bilgili A., van Es H.M., Akbas F., Durak A., Hively W.D. (2010). Visible-near infrared reflectance spectroscopy for assessment of soil properties in a semi-arid area of Turkey. J. Arid Environ..

[20] Zhang Y., Hu P., Gao J. (2011). A reflectance spectra-based approach to mapping salt fields using PCA-fused Landsat TM data. Adv. Space Res..

[21] Sidike A., Zhao S.H., Wen Y.M. (2014). Estimating soil salinity in Pingluo County of China using QuickBird data and soil reflectance spectra. Int. J. Appl. Earth Obs..

[22] Conforti M., Castrignano A., Robustelli G., Scarciglia F., Stelluti M., Buttafuoco G. (2015). Laboratory-based Vis-NIR spectroscopy and partial least square regression with spatially correlated errors for predicting spatial variation of soil organic matter content. Catena.

[23] Nawar S., Buddenbaum H., Hill J., Kozak J. (2014). Modeling and mapping of soil salinity with reflectance spectroscopy and Landsat data using two quantitative methods (PLSR and MARS). Remote Sens..

[24] Fan X., Liu Y., Tao J. (2015). Soil salinity retrieval advanced multi-spectral sensor with partial least square regression. Remote Sens..

[25] Shi X., Aspandiar M., Oldmeadow D. (2014). Using hyperspectral data and PLSR modeling to assess acid sulphate soil in subsurface. J. Soils Sediments.

[26] Tang C.S., Shi B., Liu C., Suo W.B., Gao L. (2011). Experimental characterization of shrinkage and desiccation cracking in thin clay layer. Appl. Clay Sci..

[27] Uday K.A., Singh D.N. (2013). Investigation on cracking characteristics of fine-grained soils under varied environmental conditions. Dry. Technol..

[28] Wang C., Zhang Z., Liu Y., Fan S. (2017). Geometric and fractal analysis of dynamic cracking patterns subjected to wetting-drying cycles. Soil Tillage Res..

[29] Vo T.D., Pouya A., Hemmati S., Tang A.M. (2017). Numerical modelling of desiccation cracking of clayey soil using a cohesive fracture method. Comput. Geotech..

[30] Painuli D.K., Mohanty M., Sinha N.K., Misra A.K. (2017). Crack formation in a swell-shrink soil under various managements. Agricul. Res..

[31] Abd El-Halim A.A. (2017). Image processing technique to assess the use of sugarcane pith to mitigate clayey soil cracks: Laboratory experiment. Soil Tillage Res..

[32] Smith C.W., Hadas A., Dana J., Koyumdjiskya H. (1985). Shinkage and atterberg limits in relation to other properties of principal soil types in Isreal. Geoderma.

[33] Ren J., Li X., Zhao K. (2015). Quantitative analysis of relationships between crack characteristics and properties of soda-saline soils in Songnen Plain, China. Chin. Geogr. Sci..

[34] Ren J., Li X., Zhao K., Fu B., Jiang T. (2016). Study of an on-line measurement method for the salt parameters of soda-saline soils based on the textures of cracks. Geoderma.

[35] Zhang Y., Ye W., Chen B., Chen Y., Ye B. (2016). Desiccation of NaCl-contaminated soil of earthen heritages in the Site of Yar City, Northwest China. Appl. Clay Sci..

[36] Shokri N., Zhou P., Keshmiri A. (2015). Patterns of desiccation cracks in saline bentonite layers. Transp. Porous Media.

[37] Zhang X., Chen Y., Ye W., Cui Y., Deng Y., Chen B. (2017). Effect of salt concentration on desiccation cracking behaviour of GMZ bentonite. Environ. Earth Sci..

[38] Li B., Wang Z.C., Liang Z.W., Chi C.M. (2007). Relationships between parameters of sodic saline soils in Da’an City of Jilin Province. Chin. J. Soil Sci..

[39] Zhang G., Yu Q., Wei G., Chen B., Yang Y., Hu C., Li J., Chen H. (2007). Study on the basic properties of the soda-saline soils in Songnen Plain. Hydrogeol. Eng. Geol..

[40] Li B., Wang Z., Chi C. (2006). The alkalization parameters and their changes in vertical orientation of soda solonetz soil profiles in the Da’an City, Jilin Province, China. Acta Agric. Boreali-Occident. Sin..

[41] Li B., Wang Z., Chi C. (2006). Parameters and characteristics of alkalization of sodic soil in Da’an City. J. Ecol. Rural Environ..

[42] Li B., Wang Z., Liang Z., Chi C. (2007). Relationship between salinization and alkalization of sodic soil in Da’an City. Agric. Res. Arid Areas.

[43] Xing X., Zhang W., Zhang D. (2013). Approaches and countermeasures for the sustainable development of salinization of land resources in Baicheng. J. Baicheng Norm. Univ..

[44] Zhang G., Li J., Yu Q., Zhang B., Yang R., Chen H. (2008). Effects of Salinity on shear strength of saline alkali soils in Songnen Plain. Chin. J. Geol. Hazard Control.

[45] Aksenov V.I., Kal’bergenov R.G., Leonov A.R. (2003). Strength characteristics of frozen saline soils. Soil Mechchanics Found. Eng..

[46] Jeong S.W., Locat J., Leroueil S. (2012). The effects of salinity and shear history on the rheological characteristics of illite-rich and Na-montmorillonite-rich clays. Clays Clay Miner..

[47] Farifteh J., van der Meer F., Atzberger C., Carranze E.J.M. (2007). Quantitative analysis of salt-affected soil reflectance spectra: A comparison of two adaptive methods (PLSR and ANN). Remote. Sens. Environ..

[48] Farifteh J., van der Meer F., van der Meijde M., Atzberger C. (2008). Spectral characteristics of salt-affected soils: A laboratory experiment. Geoderma.

